# Shotgun metagenomic analysis reveals taxonomic and functional alterations in the gut microbiome across prodromal and symptomatic Lewy body disease

**DOI:** 10.3389/frmbi.2026.1834726

**Published:** 2026-07-15

**Authors:** Xiaowei Zhao, Stuart J. McCarter, Vinod K. Gupta, Kiera M. Grant, Erik K. St. Louis, Kejal Kantarci, Rodolfo Savica, Max Hill, Helen E. Vuong, Christopher Staley, Bradley F. Boeve, Owen A. Ross, Levi M. Teigen, Jaeyun Sung

**Affiliations:** 1 Bioinformatics and Computational Biology Program, University of Minnesota, Rochester, MN, United States; 2 Division of Computational Biology, Department of Quantitative Health Sciences, Mayo Clinic, Rochester, MN, United States; 3 Department of Neurology, Mayo Clinic, Rochester, MN, United States; 4 Center for Sleep Medicine, Mayo Clinic, Rochester, MN, United States; 5 Microbiomics Program, Center of Individualized Medicine, Mayo Clinic, Rochester, MN, United States; 6 Division of Clinical Trials and Biostatistics, Department of Quantitative Health Sciences, Mayo Clinic, Rochester, MN, United States; 7 Department of Radiology, Mayo Clinic, Rochester, MN, United States; 8 Division of Neonatology, Department of Pediatrics, University of Minnesota, Minneapolis, MN, United States; 9 Department of Surgery, School of Medicine, University of Minnesota, Minneapolis, MN, United States; 10 Department of Neuroscience, Mayo Clinic, Jacksonville, FL, United States; 11 Department of Food Science and Nutrition, University of Minnesota, St. Paul, MN, United States

**Keywords:** dementia, gut microbiome, gut-brain axis, isolated REM sleep behavior disorder (iRBD), Lewy body disease (LBD), mild cognitive impairment (MCI), shotgun metagenomic sequencing, α-synucleinopathy

## Abstract

**Background:**

Lewy body disease (LBD) is a progressive neurodegenerative a-synucleinopathy, whereas isolated REM sleep behavior disorder (iRBD) is recognized as a prodromal stage of LBD. Although growing evidence implicates the gut–brain axis in neurodegeneration, the taxonomic and functional roles of the gut microbiome across the prodromal-to-symptomatic LBD continuum remain poorly defined.

**Methods:**

Here, we performed shotgun metagenomic sequencing on stool samples from 25 patients with LBD (10 mild cognitive impairment due to LBD [MCI-LB] and 15 dementia with Lewy bodies [DLB]), 10 individuals with iRBD, and their household matched cohabitant controls to characterize disease-associated microbial alterations while minimizing environmental confounding.

**Results:**

Despite no significant differences in global microbial diversity, we identified convergent shifts in microbial taxa, metabolic pathways, and gene families across disease stages. Both LBD and iRBD showed increased abundance of microbial taxa potentially associated with gut barrier disruption, as well as higher abundance of functional pathways related to lipopolysaccharide biosynthesis. LBD showed lower abundance of pathways related to complex carbohydrate fermentation, and both groups showed lower abundance of pathways associated with neurotransmitter-related metabolism. In particular, pathways and gene families associated with starch degradation were reduced in LBD, and those associated with histidine-to-glutamate/ GABA metabolism were reduced in both groups.

**Discussion:**

These exploratory findings represent the first high-resolution, shotgun metagenomic characterization of gut microbiome alterations across the LBD continuum, highlighting functional patterns that may serve as candidate markers of disease progression in future longitudinal and mechanistic studies.

## Introduction

Lewy body disease (LBD) refers to a spectrum of clinical conditions associated with Lewy body pathology, including mild cognitive impairment due to Lewy body disease (MCI-LB) and dementia with Lewy bodies (DLB) (McKeith et al., 2020). It is a progressive neurodegenerative disease caused by the accumulation of abnormally phosphorylated α-synuclein (Fujiwara et al., 2002). Clinically, LBD presents with a broad range of symptoms, including progressive cognitive decline, fluctuations in attention, recurrent visual hallucinations, autonomic dysfunction, parkinsonism, and REM sleep behavior disorder (RBD) (Donaghy and McKeith, 2014; Walker et al., 2015; McKeith et al., 2017). In current diagnostic criteria, the timing of cognitive and motor symptom onset helps distinguish LBD subtypes from other synucleinopathies, such as Parkinson’s disease (PD). Among these distinguishing clinical features, RBD is particularly prominent and occurs in the vast majority of individuals with LBD (Boeve et al., 1998; Ferman et al., 1999; Olson et al., 2000; Boeve et al., 2003; Iranzo et al., 2009). When RBD occurs in the absence of a diagnosed neurodegenerative disease, it is termed isolated RBD (iRBD), which is widely considered a prodromal marker of α-synucleinopathies (McKeith et al., 2005; Högl et al., 2018; Postuma and Berg, 2019). Supporting this concept, one study reported that few cases clinically diagnosed with iRBD have shown evidence for LBD pathology at post-mortem examination (Mayà et al., 2024). Taken together, these findings highlight the close link between iRBD and LBD and support their classification along a common disease continuum (Hu, 2020).

The gut-brain axis is a bidirectional communication system between the central nervous system and the gastrointestinal tract (Mayer, 2011; Mayer et al., 2015). This system operates through multiple interconnected pathways, including immune signaling, vagal and enteric neural circuits, endocrine mechanisms, and microbiota-derived metabolites and neurotransmitters (Cryan et al., 2019; Loh et al., 2024; Savulescu-Fiedler et al., 2025). Growing evidence suggests that the gut-brain axis plays a critical role in neurodegenerative diseases, with the gut microbiome increasingly implicated across several conditions (Sampson and Mazmanian, 2015; Cryan et al., 2019). In PD, for example, multiple studies have consistently reported a decreased abundance of short-chain fatty acids (SCFAs) producing bacteria and an increased prevalence of pro-inflammatory microbial taxa (Aho et al., 2021; Lubomski et al., 2022; Huang et al., 2023; Kwon et al., 2024).

Despite this growing body of work in PD, relatively few studies have examined the gut microbiome in the cognitive manifestations of LBD (i.e., DLB and MCI-LB) or in its prodromal stage, iRBD. Nishiwaki et al. analyzed stool samples from 28 patients with DLB, 26 patients with iRBD, and 147 controls (Nishiwaki et al., 2022). They reported decreased abundance in SCFAs producing bacterial genera, and increased levels of *Ruminococcus torques* and *Collinsella* in DLB compared with controls. More recently, Teigen et al. evaluated 27 LBD patients, 11 iRBD patients, and their cohabitant controls, and found no major differences in overall microbiome diversity, although the iRBD group showed a notably lower abundance of *Bacteroides (*Teigen et al., 2024).

To date, microbiome studies in LBD have relied on 16S rRNA gene amplicon sequencing, which targets a single marker gene and provides limited taxonomic resolution (primarily at the genus level) and minimal functional insight. As a result, this approach cannot fully characterize the metabolic capabilities of the microbiome or its potential contributions to disease pathology. In contrast, whole genome shotgun (WGS) metagenomic sequencing enables functional potential profiling of the microbiome by identifying microbial metabolic pathways and gene families encoded within the community (Börnigen et al., 2013). Such functional information is particularly important for LBD, as gut microbes produce SCFAs, bile acids, neurotransmitters, and other metabolites that modulate host immune responses, intestinal barrier integrity, and neural signaling (Sharon et al., 2016; Strandwitz, 2018; Parker et al., 2020). Altered microbial metabolism may therefore influence processes relevant to neurodegeneration, including neuroinflammation and oxidative stress (Erny et al., 2017; Parker et al., 2020). Therefore, studying these functional alterations provides an opportunity to identify mechanistic links between the gut microbiome and LBD pathogenesis.

In microbiome research, a high degree of inter-individual variation is commonly observed (The-Human-Microbiome-Project-Consortium, 2012; Lloyd-Price et al., 2016), driven largely by environmental factors including diet, lifestyle, and medication use (Turnbaugh et al., 2009; Zhernakova et al., 2016; Rothschild et al., 2018). Importantly, individuals who share a household tend to have more similar gut microbiota than unrelated individuals (Song et al., 2013; Rothschild et al., 2018; Tavalire et al., 2021). For this reason, the inclusion of household controls is critical for distinguishing true disease-associated microbial signatures from differences attributable to environmental or dietary variation. Without such controls, observed microbial differences may reflect lifestyle or environmental disparities rather than disease processes. Notably, only one prior LBD study—using 16S rRNA sequencing—has incorporated household-matched controls (Teigen et al., 2024), limiting the interpretability and comparability of findings from studies that did not account for this essential source of confounding.

In this study, we performed WGS metagenomic sequencing on stool samples from patients with LBD and iRBD, together with their household cohabitants. We assessed differences in microbiome diversity, taxonomic composition, and metabolic functional potential across these groups, and examined associations between microbiome features and clinical measures of cognition and motor function. By integrating high-resolution functional profiling with a household-matched design, our study aimed to identify disease-associated taxonomic and metabolic shifts across the LBD continuum while minimizing confounding from shared environmental factors.

## Materials and methods

### Participant enrollment

Participant enrollment followed procedures described in our previous work (Teigen et al., 2024). This study was approved by the Mayo Clinic Institutional Review Board (IRB: 18-011778), and informed consent was obtained from all participants. All procedures involving human participants were conducted in accordance with the ethical standards of the institutional research committee and with the Declaration of Helsinki and its later amendments. Participants were recruited through the Mayo Clinic Functional Genomics of LBD program, Mayo Clinic Alzheimer’s Disease Research Center and the North American Prodromal Synucleinopathy (NAPS) cohort. The study population included 25 patients with LBD who met research criteria for MCI-LB (n = 10) or DLB (n = 15), 10 patients with polysomnography-confirmed iRBD, as well as their household-matched (cohabitant) controls. All patients underwent a comprehensive clinical interview, neurological examination, and neuropsychological testing by sleep and behavioral neurology subspecialists and neuropsychologists. Following evaluation, a consensus clinical diagnosis of LBD or iRBD was assigned based on established clinical criteria. Since consensus diagnostic criteria for iRBD are not established, iRBD in this study was confirmed by polysomnography in the setting of normal neuropsychological testing and absence of parkinsonism. Demographic and clinical information was collected for all participants if applicable, including age, sex, dementia severity (Clinical Dementia Rating [CDR] global and sum of boxes [SB]), bedside cognitive testing score (Kokmen Short Test of Mental Status [STMS], Montreal Cognitive Assessment [MoCA]), and parkinsonism severity (Movement Disorders Society-Unified Parkinson’s Disease Rating Scale Part III [MDS-UPDRS III]).

### Stool sample collection, DNA extraction, and shotgun metagenomic sequencing

Fecal samples were collected using standardized stool specimen collection kits at the time of clinical assessment. Samples were promptly transferred to the University of Minnesota Genomics Center for DNA extraction and sequencing. Fecal DNA was extracted with the DNeasy 96 PowerSoil Pro QIAcube HT Kit (QIAGEN, Germantown, MD, USA) using the inhibitor removal technology (IRT) protocol according to the manufacturer’s instructions. DNA concentration and purity were measured with a NanoDrop-8000 UV-Vis spectrophotometer (Thermo Fisher Scientific, Wilmington, DE, USA) and a PicoGreen assay. Sequencing libraries were prepared and run on an Illumina NovaSeq 6000 using 2 × 150 bp paired-end chemistry, targeting approximately 8 million read pairs per sample.

### Quality filtration of sequenced reads

Metagenomic reads were processed using an in-house quality control pipeline. Specifically, Trimmomatic (version 0.39) (Bolger et al., 2014) was used to remove adapter and overrepresented sequences (ILLUMINACLIP: adaptor_overrepresented.fa:2:30:10:2:keepBothReads), trim low-quality bases from the start and end of reads (LEADING:3 and TRAILING:3), and discard reads shorter than 60 bp (MINLEN:60). Host-derived reads were subsequently removed by aligning to the Human Genome Reference Database (GRCh38) using Bowtie2 (version 2.5.0) (Langmead and Salzberg, 2012), followed by filtering with SAMtools (version 1.18) (Li et al., 2009).

### Taxonomic and functional profiling of stool metagenomes

Taxonomic profiling was performed using the MetaPhlAn4 (version 4.1.15) (Blanco-Míguez et al., 2023) phylogenetic clade identification pipeline with default parameters. Briefly, MetaPhlAn4 identifies microbial taxonomy and composition by detecting unique clade-specific marker genes. The marker database (mpa_vJun23_CHOCOPhlAnSGB_202403) is derived from a curated collection of 1.01 million prokaryotic reference and metagenome-assembled genomes, encompassing 26,970 species-level genome bins, 4,992 of which are taxonomically unidentified at the species level. Microbial taxa of viral origin and those labeled as unclassified or unknown were excluded from further analyses. Microbiome profiles were then normalized using total sum scaling (TSS) to obtain the relative abundances of microbial taxa. The relative abundance profiles of all detected microbial taxa are provided in **Supplementary Table 1**.

Functional profiling of microbial communities was conducted using HUMAnN3 (version 3.96) (Beghini et al., 2021) with default parameters. Briefly, HUMAnN3 first maps quality-controlled metagenomic reads to species-specific pangenomes to quantify gene families and subsequently aligns unmapped reads to the UniRef90 database to improve detection sensitivity. Gene family abundances are then used to reconstruct microbial metabolic pathways based on the MetaCyc database. Pathways classified as unmapped (i.e., reads that did not align to any gene family) or unintegrated (i.e., gene families not assigned to any known pathway) were excluded from downstream analyses. The relative abundances of the remaining pathways were normalized using TSS via the “humann_renorm_table --units relab --special n” utility in HUMAnN3. In parallel, the relative abundances of gene families were also characterized for downstream analyses. The relative abundance profiles of all detected microbial pathways are provided in **Supplementary Table 2**.

### Gut microbiome diversity analysis

Overall gut microbiome composition was evaluated by calculating both α-diversity (species-level Shannon index and richness) and β-diversity (Bray–Curtis distances between all sample pairs). The *diversity()* function from R package vegan (version 2.7.3) (Dixon, 2003) was used to calculate Shannon index and species richness based on untransformed relative abundances of microbial species in each stool metagenome. To compare α-diversity between LBD or iRBD patients and their cohabitant controls, mixed-effects linear regression models were constructed in R using the *lmer()* function from the lmerTest package (version 3.2.1) (Kuznetsova et al., 2017), with household ID included as a random effect. *P*-values less than 0.05 were considered statistically significant. For β-diversity analysis, principal coordinate analysis (PCoA) was performed using Bray–Curtis dissimilarities calculated on arcsine square-root-transformed relative abundances of microbial species and pathways identified by MetaPhlAn4 and HUMAnN3. This analysis was implemented using the R packages ade4 (version 1.7.24) (Chessel et al., 2004) and vegan (version 2.7.3) (Dixon, 2003). All statistical analyses described above were conducted in R (version 4.5.3).

### PERMANOVA on taxonomic composition of microbial communities

A permutational multivariate analysis of variance (PERMANOVA) was conducted on the Bray–Curtis dissimilarity matrix using the *adonis2()* function from the R package vegan (version 2.7.3) (Dixon, 2003). *P*-values for the pseudo-F statistic were calculated based on 999 permutations to assess the proportion of variance in gut microbial community composition explained by case-control status and patient characteristics such as age, sex, and BMI. For comparisons between LBD or iRBD patients and their cohabitant controls, permutations were constrained within households using the strata option to account for paired study design. The output from PERMANOVA was used to estimate the percentage of variance in microbiome composition attributable to case-control status and its statistical significance. *P*-values less than 0.05 were considered statistically significant. All statistical analyses described above were conducted in R (version 4.5.3).

### Differential abundance and prevalence analysis

Microbial taxonomic and functional features (species, pathways, and gene families) were preprocessed using two filtering steps prior to differential analysis. First, to reduce the influence of extremely low-abundance features that may represent noise or technical artifacts, relative abundance values below a predefined threshold were set to zero based on rank-abundance plots (**Supplementary Figures 1A–C**). Specifically, presence/absence thresholds for each feature type were determined empirically by ranking all features by their relative abundance and identifying the elbow point of the resulting rank-abundance curve, beyond which the distribution drops sharply toward the detection limit. This approach yielded thresholds of 10^–4.7^,10^–4.5^, and 10^–7^ for microbial species, metabolic pathways, and gene families, respectively (**Supplementary Figure 1**). Second, to reduce extreme data sparsity inherent to microbiome data, microbial features were filtered using a 10% prevalence cutoff. That is, taxonomic or functional features detected in fewer than 10% of samples within each group comparison were excluded prior to downstream differential analyses. The 10% prevalence threshold was chosen based on established practice in microbiome studies (Nearing et al., 2022; Yang and Chen, 2022), where features present in fewer than 10% of samples are considered too sparse to yield reliable statistical estimates. Mixed-effects linear regression models were applied to arcsine square-root-transformed relative abundance data to identify differentially abundant features (species, pathways, and gene families) between LBD or iRBD patients and their cohabitant controls. The models included age and BMI as fixed effects and household ID as a random effect. Age and BMI were included as covariates given their well-documented influence on gut microbiome composition (Turnbaugh and Gordon, 2009; Ghosh et al., 2020). Although antibiotic use and constipation are also recognized modulators of the gut microbiome (Zhu et al., 2014; Iizumi et al., 2017), these variables could not be included in the models due to data availability constraints. Antibiotic use data were missing for six participants across the LBD and control groups, and constipation status was only available for LBD and iRBD participants but not for their cohabitant controls. All models were fitted in R using the *lmer()* function from the lmerTest package (version 3.2.1) (Kuznetsova et al., 2017). To quantify the magnitude of microbial species and pathway relative abundance differences between patients and their cohabitant controls, Cohen’s *d* was calculated for each microbial species and pathway feature. All analyses were performed in R using the *cohens_d()* function from the effectsize package (version 1.0.2) (Ben-Shachar et al., 2020). Differentially prevalent microbial features were identified using Fisher’s exact test. All *P*-values reported here were unadjusted for multiple comparisons. Unadjusted *P*-values less than 0.05 were considered statistically significant. All statistical analyses described above were conducted in R (version 4.5.3).

### Correlations between clinical characteristics and microbial features

To investigate correlation between microbial feature abundance and clinical measurements (functional impairment [CDR-SB], cognitive performance [MoCA and STMS], and motor severity [MDS-UPDRS III]), Spearman partial correlation analyses were performed separately for the LBD (n = 25) and iRBD (n = 10) groups. To reduce sparsity and improve the robustness of the analysis, untransformed relative abundances of microbial features (species and pathways) were filtered by prevalence; only features present in more than 50% of samples within each group were retained for downstream analysis.

For each retained feature, the Spearman partial correlation coefficient *ρ* was estimated against each clinical measurement, adjusting for age and BMI as covariates using the *pcor.test()* function from the ppcor package (version 1.1) (Kim, 2015) in R. Features with |*ρ*| > 0.4 and unadjusted *P* < 0.05 were considered statistically significant and retained for further analysis.

Given the small sample sizes, particularly in the iRBD group, a resampling-based validation was applied to all features meeting the above criteria to evaluate the robustness of each observed correlation. An empirical *P*-value was derived from a permutation null distribution constructed as follows: in each of 1,000 iterations, a random subsample of up to n−1 observations (80–96% for LBD; 80–90% for iRBD) of the available observations was drawn without replacement, the clinical measurement labels were randomly permuted within the subsample to break any true correlation, and a Spearman partial correlation adjusting for age and BMI was computed. The empirical *P*-value was defined as the proportion of null |*ρ*| values that equaled or exceeded the observed |*ρ*| from the full sample. All *P*-values reported here are unadjusted for multiple comparisons. Features with an unadjusted empirical *P* < 0.05 were considered to show robust correlations with the clinical characteristic of interest. All statistical analyses described above were conducted in R (version 4.5.3).

## Results

### Participant demographics

**Figure 1:**
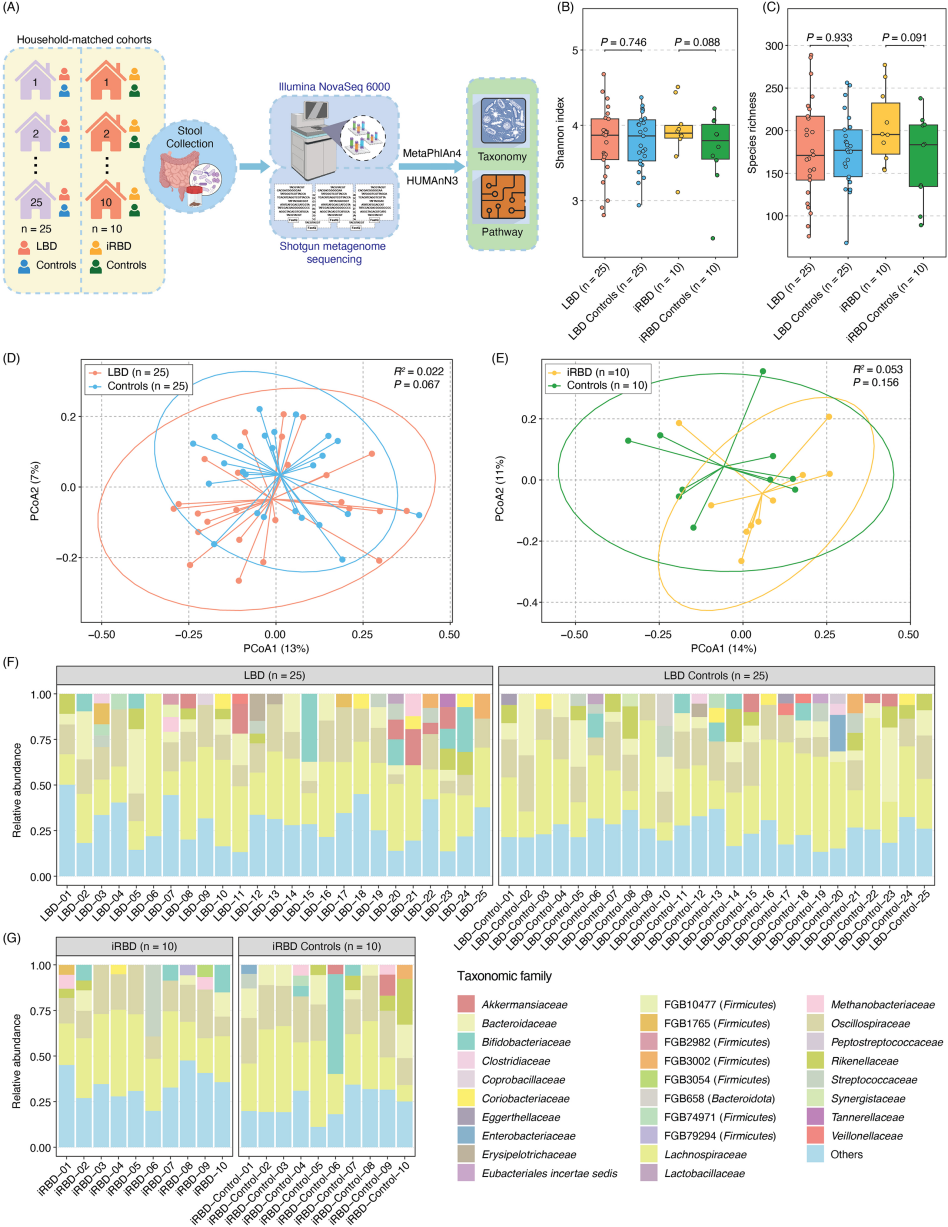
Study design overview and gut microbiome community comparisons. **(A)** Stool samples were collected from patients with LBD (n = 25), iRBD (n = 10), and their cohabitant controls. Whole genome shotgun (WGS) metagenome sequencing was performed on all samples using the Illumina NovaSeq 6000 platform. Microbial taxonomic and functional profiling were conducted using MetaPhlAn4 and HUMAnN3, respectively. Icons used here were created with BioRender.com. **(B–C)** Boxplots show the species-level Shannon index and richness between LBD or iRBD patients and their cohabitant control groups. Statistical comparisons between patients and controls were performed using mixed-effects linear regression models with household ID as a random effect (to account for intra-household correlation). **(D–E)** Principal coordinate analysis (PCoA) ordination plots based on species-level Bray–Curtis dissimilarity showed no clear separation by case-control status, and permutational multivariate analysis of variance (PERMANOVA) tests indicated that disease diagnosis explained 2.2% and 5.3% of the variance in overall microbial community. **(F–G)** Stacked bar plots showing the relative abundance of taxonomic families in LBD, iRBD, and their cohabitant controls. Families with a mean relative abundance below 5% or unclassified taxa were grouped into “Others”. Each bar represents one sample.

This study includes stool samples from 25 patients with LBD, 10 patients with iRBD, and their cohabitant controls (**Figure 1A**). Participant demographic and clinical characteristics are shown in **Table 1**. LBD patients ranged in age from 46 to 73 years (median = 68), and iRBD patients from 56 to 76 years (median = 68). Both groups were slightly older than their cohabitant controls; however, the differences were not statistically significant (*P* = 0.099 and 0.149, respectively; Mann–Whitney U test). Male sex accounted for 84% and 90% of LBD and iRBD patients, respectively, indicating a strong male predominance in both groups (*P* ≤ 0.001 for both; Fisher’s exact test). Although five iRBD patients had a global CDR score of 0.5 based on reported degree of cognitive impairment, their neuropsychometric performance was within normal limits and they were therefore classified as having iRBD rather than MCI-LB.

**Table 1:** Demographic and clinical characteristics of the study participants.

Patient characteristics	LBD^a^			iRBD^b^		
	Patients (*n* = 25)	Cohabitant controls (*n* = 25)	*P-*value^#^	Patients (*n* = 10)	Cohabitant controls (*n* = 10)	*P-*value^#^
Age						
Median [Q1, Q3]^c^	68.0 [64.0, 73.0]	64.0 [59.0, 71.0]	0.099	68.0 [63.5, 69.8]	63.0 [57.5, 65.5]	0.149
Sex						
Female, *n* (%)	4 (16)	23 (92)	< 0.001	1 (10)	9 (90)	0.001
Male, *n* (%)	21 (84)	2 (8)		9 (90)	1 (10)	
BMI						
Median [Q1, Q3]	26.4 [25.1, 31.2]	26.6 [25.1, 29.3]	0.288	28.9 [26.9, 29.4]	27.5 [24.2, 29.5]	0.533
Not available	2	10		0	3	
Global CDR^d^, *n* (%)						
0	0 (0)	N/A		5 (50)	N/A	
0.5	16 (64)			5 (50)		
1	8 (32)			0 (0)		
2	0 (0)			0 (0)		
3	1 (4)			0 (0)		
CDR-SB^e^, *n* (%)						
0	0 (0)	N/A		5 (50)	N/A	
0.5–4.0	15 (60)			5 (50)		
4.5–9.0	9 (36)			0 (0)		
9.5–15.5	0 (0)			0 (0)		
16.0–18.0	1 (4)			0 (0)		
MDS-UPDRS III^f^						
Median [Q1, Q3]	19.0 [1.0, 26.0]	N/A		0.0 [0.0, 2.5]	N/A	
MoCA^g^						
Median [Q1, Q3]	21.0 [16.8, 27.3]	N/A		25.5 [25.0, 28.5]	N/A	
Not available	1			0		
STMS^h^						
Median [Q1, Q3]	31.0 [27.0, 34.0]	N/A		36.5 [36.0, 37.8]	N/A	
Visual hallucinations, *n* (%)	9 (36)	N/A		0 (0)	N/A	
Cognitive fluctuations, *n* (%)	10 (40)	N/A		0 (0)	N/A	
Parkinsonism, *n* (%)	18 (72)	N/A		1^^^ (10)	N/A	
REM sleep behavior disorder, *n* (%)	24 (96)	N/A		10 (100)	N/A	
Antibiotic use in the last 6 months, *n* (%)						
*n* (%)	8 (32)	4 (16)		1 (4)	2 (8)	
Not available	4	2		1	1	
Constipation in the last 6 months, *n* (%)						
*n* (%)	20 (80)	N/A		5 (50)	N/A	
Not available	1			0		

^a^LBD, Lewy body disease; ^b^iRBD, isolated REM sleep behavior disorder; ^c^Upper and lower quartiles; ^d^Global CDR, Global Clinical Dementia Rating; ^e^CDR-SB, Clinical Dementia Rating Scale Sum of Boxes; ^f^MDS-UPDRS III, Movement Disorder Society-Unified Parkinson’s Disease Rating Scale Part III score; ^g^MoCA, Montreal Cognitive Assessment test score; ^h^STMS, Short Test of Mental Status score; #Mann-Whitney *U* test and Fisher’s exact test was used to test for the statistical significance of age and sex, respectively; and mixed-effects linear regression was used to test for the statistical significance of BMI accounting for household as a random effect; N/A, not applicable or not available. ^One iRBD participant had mild bradykinesia suggestive of very mild parkinsonism but without other cardinal manifestations that would meet criteria for PD.

### Overall comparisons of gut microbiome communities between LBD or iRBD patients and their cohabitant controls

Species-level Shannon index and richness did not show significant differences between LBD or iRBD patients and their cohabitant controls (**Figures 1B, C**). This suggests that overall microbial α-diversity at the species level was similar between disease and control groups. For β-diversity, PERMANOVA analysis showed that disease diagnosis explained 2.2% and 5.3% of the total variance in gut microbial community composition in LBD and iRBD cohorts, respectively; however, the variance explained by case-control status was not significant in either cohort (*P* = 0.067 and 0.156; **Figures 1D, E**). α- and β-diversity analysis based on metabolic pathway relative abundance also showed no significant differences between disease groups and their cohabitant controls (**Supplementary Figures 2A–D**).

We also performed PERMANOVA analysis to evaluate other patient characteristics that might contribute to variance in gut microbial communities of LBD and iRBD cohorts. In seperate models, age, sex, and BMI explained 2.2%, 2.0%, and 2.6%, respectively, of the total variance in gut microbial communities among individuals from the LBD cohort, and 5.0%, 4.7%, and 5.3%, respectively, among those from the iRBD cohort (**Table 2**). However, none of these variables explained a significant amount of variance in either cohort (**Table 2**).

**Table 2:** Patient characteristics contributing to the variance in gut microbial community composition.

Cohort	Patient characteristics	Variance explained (%)	*P*-value^#^
LBD (*n* = 25)	Case-control status	2.2	0.067
	Age	2.2	0.076
	Sex	2.0	0.114
	BMI	2.6	0.235
iRBD (*n* = 10)	Case-control status	5.3	0.156
	Age	5.0	0.567
	Sex	4.7	0.339
	BMI	5.3	0.378

^#^Permutational Multivariate Analysis of Variance (PERMANOVA) was used to test for the association between the corresponding patient characteristic and compositional variance within the gut microbiome.

The relative abundance of microbial taxonomic families in the gut microbiomes of individuals with LBD or iRBD and their cohabitant controls is shown in **Figures 1F, G**. *Lachnospiraceae* was the most abundant family in all four groups (LBD, LBD controls, iRBD, and iRBD controls) (median relative abundance = 0.304, 0.312, 0.273, 0.307, respectively). *Oscillospiraceae* and *Bacteroidaceae* were also abundant across all groups. The median relative abundance of *Oscillospiraceae* was 0.160, 0.168, 0.181, and 0.232 in the LBD, LBD controls, iRBD, and iRBD controls, respectively; while *Bacteroidaceae* had median values of 0.050, 0.087, 0.000, and 0.085 in the same groups. Some families, such as *Akkermansiaceae* and *Bifidobacteriaceae*, were present in high abundance in a few individuals but absent in others. Overall, the microbial profiles at the family level indicate clear inter-individual heterogeneity.

### Differentially abundant and prevalent gut microbiome features between LBD patients and their cohabitant controls

**Figure 2:**
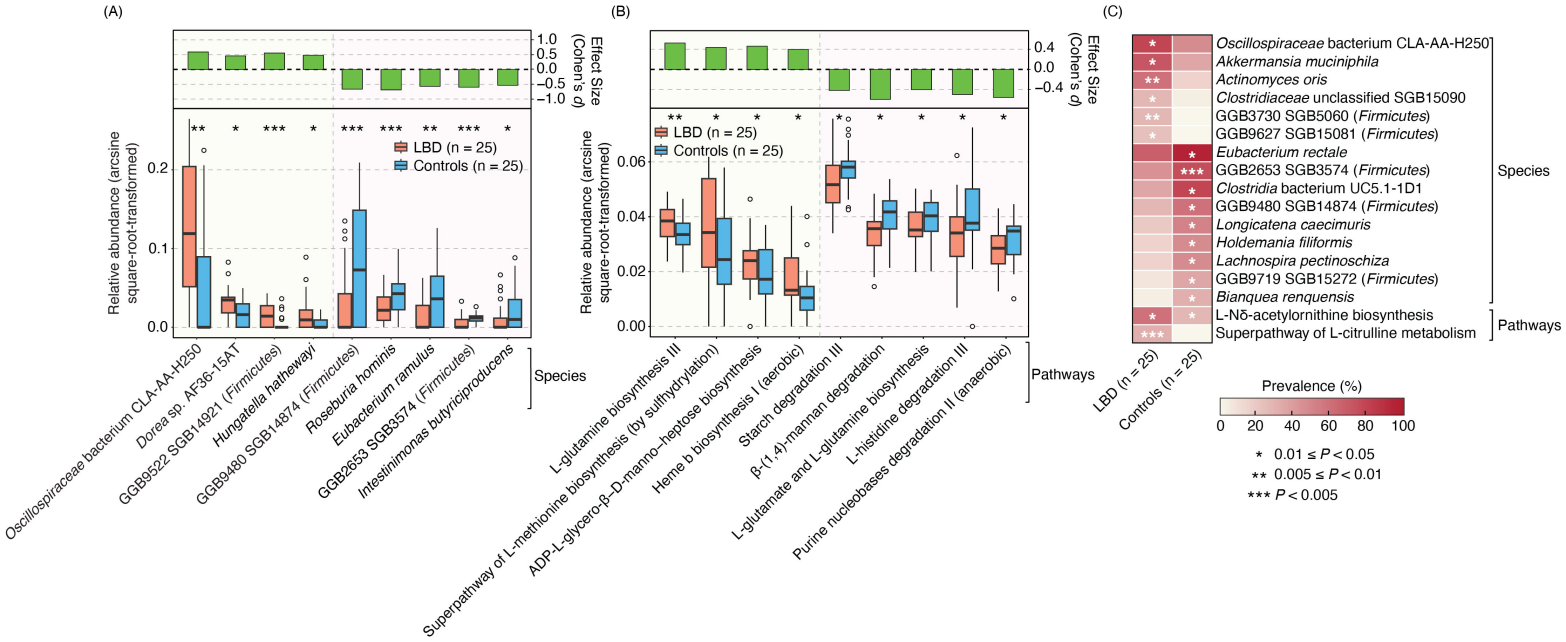
Differentially abundant and prevalent gut microbiome features between patients with LBD and their cohabitant controls. Boxplots show the top microbial species **(A)** and metabolic pathways **(B)**, up to five per panel if available. These features were selected based on unadjusted *P*-values and showed significant differences in relative abundance between LBD and control groups. *P*-values were obtained from mixed-effects linear regression models adjusting for age and BMI, with household ID included as a random effect. Bar plots above each box represent the effect size (Cohen’s *d*) between LBD and control groups; positive values indicate features more abundant in LBD, while negative values indicate features more abundant in controls. **(C)** Heatmap shows microbial species and metabolic pathways that were significantly different in prevalence between groups. Color intensity reflects the proportion of individuals in each group carrying the corresponding feature. *P*-values were obtained from Fisher’s exact test. *0.01 ≤ *P* < 0.05; **0.005 ≤ *P* < 0.01; ***P < 0.005. All *P*-values reported are unadjusted for multiple comparisons.

Although no significant differences in α- and β-diversity were observed between LBD and their cohabitant controls, differential abundance and prevalence analysis of microbial taxa and metabolic pathways revealed several compositional differences between disease and cohabitant control groups. Filtering with the prevalence cut-off (described in **Materials and Methods**) yielded a total of 492 microbial species and 433 metabolic pathways for downstream differential analyses. Using a mixed-effects linear regression model adjusted for age and BMI, with household ID as a random effect, we identified that four species were significantly more abundant in LBD, while nine were elevated in controls (unadjusted *P* < 0.05; median |Cohen’s *d*| = 0.533; range: 0.368–0.692; **Supplementary Figure 3A**; **Supplementary Table 3**). Microbial species that were more abundant in LBD included *Oscillospiraceae* bacterium CLA-AA-H250, *Dorea* sp. AF36-15AT, GGB9522 SGB14921 (*Firmicutes*), and *Hungatella hathewayi*, whereas GGB9480 SGB14874 (*Firmicutes*), *Roseburia hominis*, *Eubacterium ramulus*, GGB2653 SGB3574 (*Firmicutes*), and *Intestinimonas butyriciproducens* were more abundant in controls (**Figure 2A**). Applying the same model to microbial metabolic pathways, we identified four pathways with significantly higher relative abundance in LBD and six pathways elevated in controls (unadjusted *P* < 0.05; median |Cohen’s *d*| = 0.451; range: 0.397–0.593; **Supplementary Figure 3B**, **Supplementary Table 4**). Pathways enriched in LBD included L-glutamine biosynthesis III, the superpathway of L-methionine biosynthesis by sulfhydrylation, ADP-L-glycero-β-D-manno-heptose biosynthesis, and heme b biosynthesis I (aerobic), whereas pathways enriched in the control group included starch degradation III, β-(1,4)-mannan degradation, L-glutamate and L-glutamine biosynthesis, L-histidine degradation III, and purine nucleobase degradation II (anaerobic) (**Figure 2B**). Full results for differentially abundant microbial species and pathways, including FDR-adjusted *P*-values, are provided in **Supplementary Tables 3** and **4**.

In addition to differentially abundant microbial features, we found that six microbial species and two metabolic pathways were significantly more prevalent in LBD, while nine microbial species were more prevalent in controls (unadjusted *P* < 0.05, Fisher’s exact test; **Figure 2C**; **Supplementary Tables 5** and **6**). When viewed together with the differential abundance results, several microbial species showed consistent patterns. *Oscillospiraceae* bacterium CLA-AA-H250 was not only more abundant in LBD but also present in a greater number of LBD individuals. Similarly, species such as *Eubacterium rectale*, GGB2653 SGB3574 (*Firmicutes*), GGB9480 SGB14874 (*Firmicutes*), and *Longicatena caecimuris* exhibited both higher relative abundance and prevalence in the control group, which indicates these taxa may serve as robust microbial signatures distinguishing LBD from controls. Full results for differentially prevalent microbial species and pathways, including FDR-adjusted *P*-values, are provided in **Supplementary Tables 5** and **6**.

### Differentially abundant and prevalent gut microbiome features between iRBD patients and their cohabitant controls

**Figure 3:**
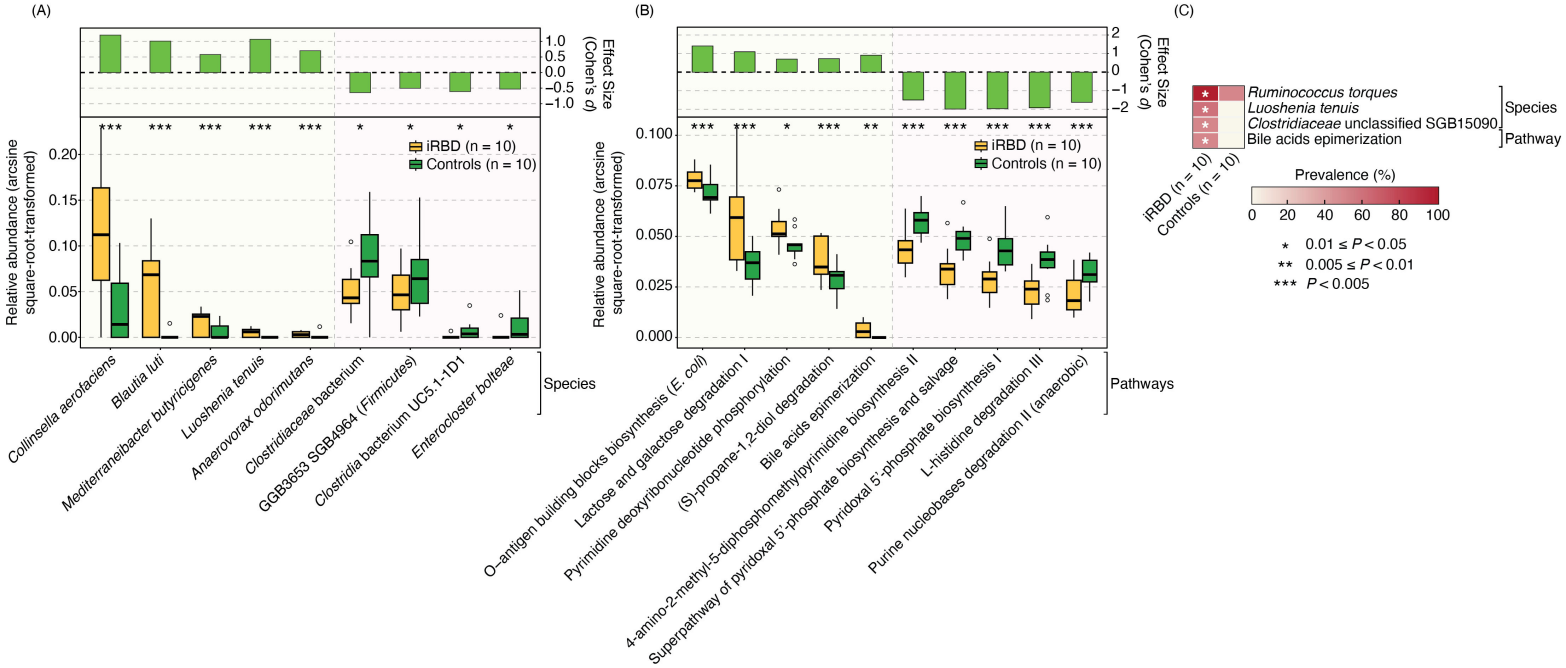
Differentially abundant and prevalent gut microbiome features between patients with iRBD and their cohabitant controls. Boxplots show the top microbial species **(A)** and metabolic pathways **(B)**, up to five per panel if available. These features were selected based on unadjusted *P*-values and showed significant differences in relative abundance between iRBD and control groups. *P*-values were obtained from mixed-effects linear regression models adjusting for age and BMI, with household ID included as a random effect. Bar plots above each box represent the effect size (Cohen’s *d*) between iRBD and control groups; positive values indicate features more abundant in iRBD, while negative values indicate features more abundant in controls. **(C)** Heatmap shows microbial species and metabolic pathways that were significantly different in prevalence between groups. Color intensity reflects the proportion of individuals in each group carrying the corresponding feature. *P*-values were obtained from Fisher’s exact test. *0.01 ≤ *P* < 0.05; **0.005 ≤ *P* < 0.01; ***P < 0.005. All *P*-values reported are unadjusted for multiple comparisons.

After applying the prevalence cut-off (described in Materials and Methods), 589 microbial species and 439 metabolic pathways remained for further analysis. With a mixed-effects linear regression model adjusted for age, BMI, and household ID, we found 23 microbial species that were significantly more abundant in iRBD; and four species showing higher abundance in controls (unadjusted *P* < 0.05; median |Cohen’s *d*| = 0.696; range: 0.432–1.278; **Supplementary Figure 3C**; **Supplementary Table 7**). Microbial species that were more abundant in iRBD included *Collinsella aerofaciens*, *Blautia luti*, *Mediterraneibacter butyricigenes*, *Luoshenia tenuis*, and *Anaerovorax odorimutans*, whereas the control group showed higher abundance of *Clostridiaceae* bacterium, GGB3653 SGB4964 (*Firmicutes*), *Clostridia* bacterium UC5.1-1D1, and *Enterocloster bolteae* (**Figure 3A**). Metabolic pathway analysis using the same approach identified 26 pathways that had significantly higher relative abundance in iRBD, as well as 39 pathways being more abundant in controls (unadjusted *P* < 0.05; median |Cohen’s *d*| = 0.755; range: 0.008–1.984; **Supplementary Figure 3D**; **Supplementary Table 8**). The top five pathways enriched in iRBD included O-antigen building blocks biosynthesis (*E. coli*), Lactose and galactose degradation I, Pyrimidine deoxyribonucleotide phosphorylation, (S)-propane-1,2-diol degradation, and Bile acids epimerization, while the control group showed greater abundance of pathways such as 4-amino-2-methyl-5-diphosphomethylpyrimidine biosynthesis II, Superpathway of pyridoxal 5’-phosphate biosynthesis and salvage, Pyridoxal 5’-phosphate biosynthesis I, L-histidine degradation III, and Purine nucleobases degradation II (anaerobic) (**Figure 3B**). Full results for differential abundance analysis of microbial species and pathways, including FDR-adjusted *P*-values, are provided in **Supplementary Tables 7** and **8**.

In addition to differentially abundant microbial features, we identified three microbial species and one metabolic pathway that were significantly more prevalent in iRBD patients compared with their cohabitant controls (unadjusted *P* < 0.05, Fisher’s exact test; **Figure 3C**; **Supplementary Tables 9** and **10**). When integrating results from abundance and prevalence analyses, several features demonstrated consistent group-wise differences. Specifically, *Luoshenia tenuis*, *Clostridiaceae* unclassified SGB15090, and the Bile acids epimerization pathway were not only significantly more abundant in the iRBD group but also present in a greater proportion of iRBD individuals, which suggests these features as potential microbial signatures associated with iRBD. Full results for differential prevalence analysis of microbial species and pathways, including FDR-adjusted *P*-values, are provided in **Supplementary Tables 9** and **10**.

### Microbial features characterizing the disease continuum of Lewy body disease

Several microbial features showed consistent directional changes in both LBD and iRBD compared with their cohabitant controls, although statistical significance was reached only in the LBD cohort (**Supplementary Figure 4**). For example, *Oscillospiraceae* bacterium CLA-AA-H250, *Akkermansia muciniphila*, *Actinomyces oris*, GGB3730 SGB5060 (*Firmicutes*), and GGB9627 SGB15081 (*Firmicutes*) were more prevalent in both disease groups, but these increases were statistically significant only in LBD. Conversely, *Clostridia* bacterium UC5.1-1D1, *Longicatena caecimuris*, and GGB9719 SGB15272 (*Firmicutes*) were more commonly detected in cohabitant controls, again with significance limited to LBD. Similar patterns were observed for two metabolic pathways, Superpathway of L-citrulline metabolism and L-Nδ-acetylornithine biosynthesis, which were more prevalent in both disease groups but reached statistical significance only in LBD. Together, these findings suggest that several microbial alterations may be present during the prodromal phase (iRBD) but intensify or become more consistently detectable as the disease progresses to overt LBD. This progression-like pattern supports the concept of a microbial signature that evolves along the Lewy body disease continuum.

### Shared microbial features between LBD and iRBD compared with cohabitant controls

To identify gut microbial alterations common across the LBD disease spectrum, we examined features that were altered in the same direction in both LBD and iRBD relative to their cohabitant controls. At the species level, Clostridiaceae unclassified SGB15090 was significantly more prevalent in both LBD and iRBD groups (unadjusted *P* < 0.05; **Figures 2C**, **3C**). At the genus level, *Bacteroides* was significantly less abundant in both LBD and iRBD, consistent with patterns previously reported in α-synucleinopathies (Minato et al., 2017; Petrov et al., 2017; Teigen et al., 2024) (unadjusted *P* < 0.05; **Supplementary Figure 5**; **Supplementary Tables 11** and **12**). At the pathway level, the relative abundances of L-histidine degradation III, Purine nucleobases degradation II (anaerobic), and L-glutamate and L-glutamine biosynthesis were consistently lower in both disease groups compared with controls (unadjusted *P* < 0.05). Together, these shared alterations suggest common taxonomic and metabolic disruptions that may be present in the prodromal stage (iRBD) through the symptomatic stage of LBD.

### Decreased metabolic potential for starch degradation in LBD compared with cohabitant controls

Among pathways that were less abundant in LBD compared with controls, Starch degradation III displayed the strongest statistical significance (unadjusted *P* = 0.012, mixed-effects linear regression; **Figure 2B**). Its reduced abundance in LBD suggests a diminished microbial capacity for starch breakdown, which could contribute to lower SCFAs production.

**Figure 4:**
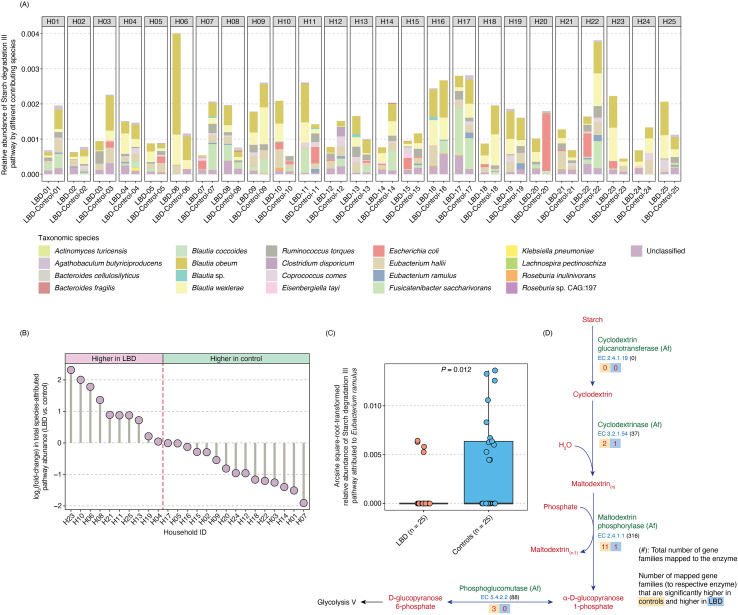
Comparison of the starch degradation III pathway between LBD and their cohabitant controls. **(A)** Species-level contribution to the starch degradation III pathway. Stacked bar plots for all 25 households show the microbial species contributing to the starch degradation III pathway in paired LBD and control samples. Contributions from unmapped or unintegrated pathways were excluded. **(B)** Change in total pathway contribution in LBD relative to control within each household. LBD samples showed higher pathway contributions in ten households, whereas controls showed higher contributions in fifteen households. **(C)** Boxplot of the arcsine square-root-transformed relative abundance of starch degradation III attributed specifically to *Eubacterium ramulus*, which was significantly higher in controls compared with LBD patients (unadjusted *P* = 0.012). **(D)** Schematic diagram of the starch degradation III pathway highlights four key enzymatic steps. Numbers in parentheses indicate the total gene families mapped to each enzyme. Yellow and blue boxes denote gene families with higher abundance in controls and LBD, respectively. All *P*-values reported are unadjusted for multiple comparisons.

Using HUMAnN3, we quantified species-attributed contributions to the Starch degradation III pathway in LBD patients and their cohabitant controls (**Figure 4A**). Specifically, this represents the proportion of total pathway abundance that can be assigned to individual microbial species based on their encoded gene families, thereby quantifying how much each species functionally contributes to the pathway. Substantial inter-individual variability was observed in the type of taxa contributing to the pathway, as well as in their relative contributions; however, *Blautia obeum* and *Blautia wexlerae* consistently accounted for the largest proportion of pathway activity across groups.

When comparing total species-attributed contributions within each household, the controls exhibited higher overall pathway contribution in 15 households (60%), whereas LBD patients showed higher contribution in only 10 households (40%) (**Figure 4B**). This household-level pattern highlights a consistent shift toward reduced starch-degrading capacity in LBD, even when controlling for shared environmental and dietary factors. At the individual species level, *Eubacterium ramulus* was the only species that contributed significantly more to this pathway in controls than in LBD patients (unadjusted *P* = 0.012; **Figure 4C**).

Finally, mapping gene families to specific enzymatic steps within the pathway indicated that three of the four steps had a greater number of gene families with higher abundance in controls than in LBD patients (**Figure 4D**). This step-level reduction, when considered alongside lower pathway abundance and diminished contributions from key carbohydrate-utilizing taxa, such as *Eubacterium ramulus*, points to a coordinated loss of functional capacity across multiple layers of the pathway. Together, these multi-layered analyses (pathway abundance, species-level contributions, and gene family distributions) provide convergent evidence for a reduction in microbial starch degradation potential in LBD, which may contribute to impaired fermentation of complex carbohydrates and reduced SCFAs availability.

### Decreased metabolic potential for L-histidine degradation in both LBD and iRBD compared with cohabitant controls

**Figure 5:**
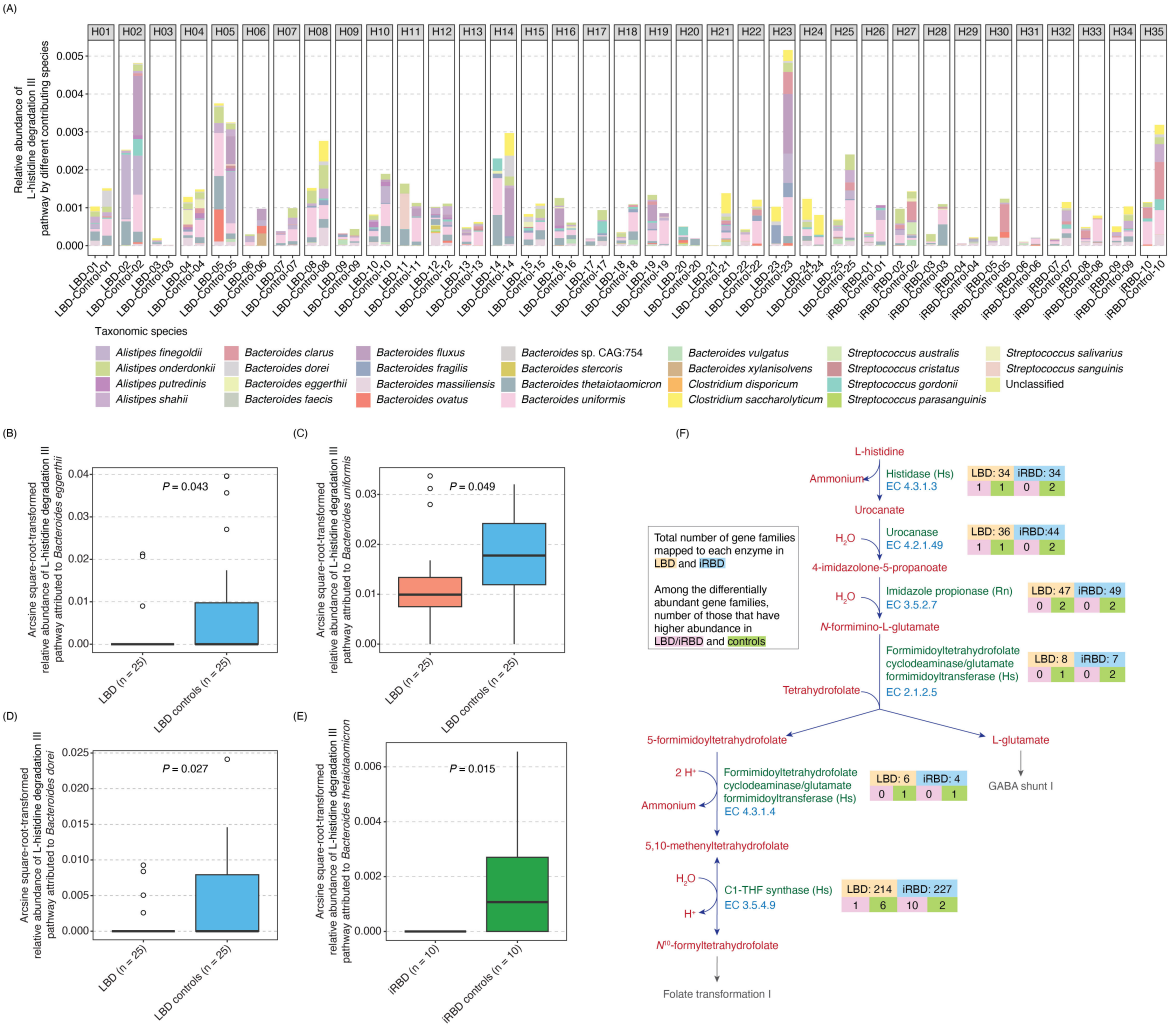
Comparison of the L-histidine degradation III pathway between LBD or iRBD and their cohabitant controls. **(A)** Species-level contribution to the L-histidine degradation III pathway in paired LBD or iRBD and their cohabitant controls. Stacked bar plots for all 35 households show the microbial species contributing to the L-histidine degradation III pathway in paired LBD or iRBD and their controls. Contributions from unmapped or unintegrated pathways were excluded. **(B–D)** Boxplot of the arcsine square-root-transformed relative abundance of the L-histidine degradation III pathway contributed by *Bacteroides eggerthii, Bacteroides uniformis, and Bacteroides dorei*, which were significantly higher in controls than in LBD patients. **(E)** Similarly, *Bacteroides thetaiotaomicron* contributed to higher relative abundance of this pathway in controls than in iRBD patients. **(F)** Schematic diagram of the L-histidine degradation III pathway highlights key enzymatic steps and intermediate metabolites. Numbers in yellow boxes indicate the total number of gene families mapped to each enzyme in LBD patients and controls; numbers in blue boxes indicate totals for iRBD patients and controls. Pink boxes denote the number of differentially abundant gene families with higher abundance in the disease group, while green boxes indicate those with higher abundance in controls. All *P*-values reported are unadjusted for multiple comparisons.

The L-histidine degradation III pathway was one of the few metabolic pathways consistently reduced in both LBD and iRBD compared with their cohabitant controls. Given its role in producing L-glutamate, which is a microbial precursor for γ-aminobutyric Acid (GABA) synthesis, we further examined its contributing taxa and associated gene families to better characterize its functional decline. This pathway was significantly more abundant in cohabitant controls than in both LBD and iRBD groups (unadjusted *P* = 0.027 and unadjusted *P* = 5.47×10^–5^, respectively). Household-level stacked bar plots revealed substantial inter-individual variability in contributing species; however, *Bacteroides uniformis* emerged as the most consistently detected contributor across all groups (**Figure 5A**).

At the species level, several *Bacteroides* taxa showed significantly reduced contributions to L-histidine degradation III in disease groups. Contributions from *Bacteroides eggerthii*, *Bacteroides uniformis*, and *Bacteroides dorei* were significantly lower in LBD (unadjusted *P* < 0.05; **Figures 5B–D**), while *Bacteroides thetaiotaomicron* showed a reduction in iRBD (unadjusted *P* < 0.05; **Figure 5E**). These patterns parallel the decreased abundance of multiple *Bacteroides* species observed in both LBD and iRBD, suggesting that loss of *Bacteroides*-mediated metabolism may underlie the overall reduction in L-histidine degradation capacity.

The L-histidine degradation III pathway comprises several enzymatic steps that generate intermediates feeding into glutamate and downstream GABA production (**Figure 5F**). Mapping gene families to these steps demonstrated a broad reduction in functional potential across the pathway; at nearly every enzymatic step, a greater number of gene families showed higher abundance in controls than in either disease group. In iRBD, reductions were evident in earlier steps of the pathway, whereas in LBD, deficits extended further downstream, particularly within folate-dependent transformations (e.g., MTHFD1). This progressive erosion of gene family abundance from iRBD to LBD suggests a stepwise decline in microbial histidine catabolism along the disease continuum, potentially reducing microbial contributions to glutamate and GABA availability in the gut.

**Figure 6:**
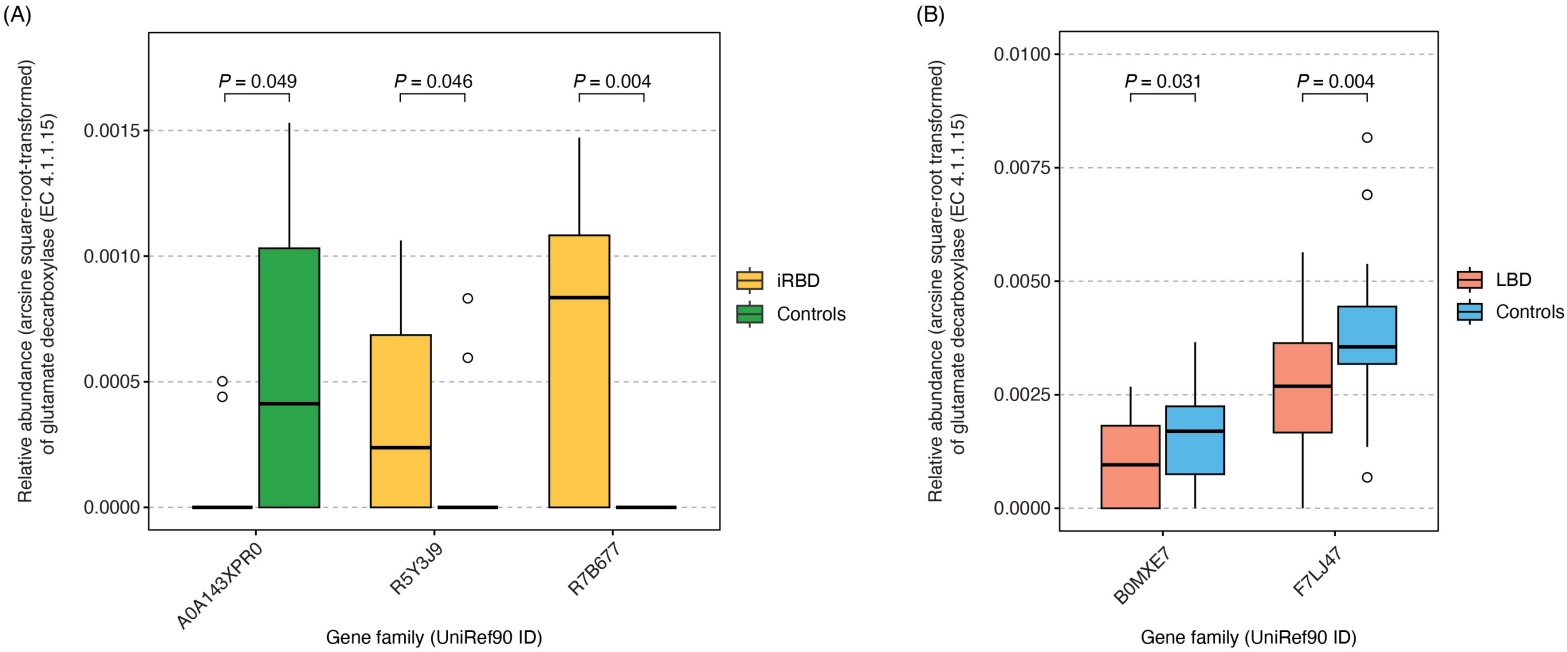
Relative abundance of gene families (UniRef90 database) mapped to glutamate decarboxylase (EC 4.1.1.15). **(A)** Boxplots show the arcsine square-root-transformed relative abundance of three gene families associated with glutamate decarboxylase in iRBD patients and their cohabitant controls. One of the three gene families exhibited significantly higher abundance in controls. **(B)** Boxplots show two glutamate decarboxylase-associated gene families that differed significantly between LBD patients and their cohabitant controls, with both gene families showing higher abundance in controls. All *P*-values reported are unadjusted for multiple comparisons.

One of the final products of the L-histidine degradation III pathway is L-glutamate, which can enter the GABA synthesis pathway (GABA shunt) and be converted to GABA, which is one of the main inhibitory neurotransmitters of the brain. The key enzyme catalyzing this conversion is glutamate decarboxylase (EC 4.1.1.15). To investigate the microbial potential for this enzymatic step, we compared the abundance of gene families mapped to glutamate decarboxylase between LBD or iRBD and their cohabitant controls. In LBD, two differentially abundant gene families mapped to this enzyme showed higher abundance in controls, whereas in iRBD, one of three differentially abundant gene families showed higher abundance in controls (**Figures 6A, B**). Taken together, these findings suggest a progressive reduction in microbial glutamate decarboxylase potential from the prodromal stage (iRBD) to the symptomatic stage (LBD), which may reflect declining microbial capacity to support gut-derived GABA production along the disease continuum.

### Correlation between clinical measures and microbial features

**Figure 7:**
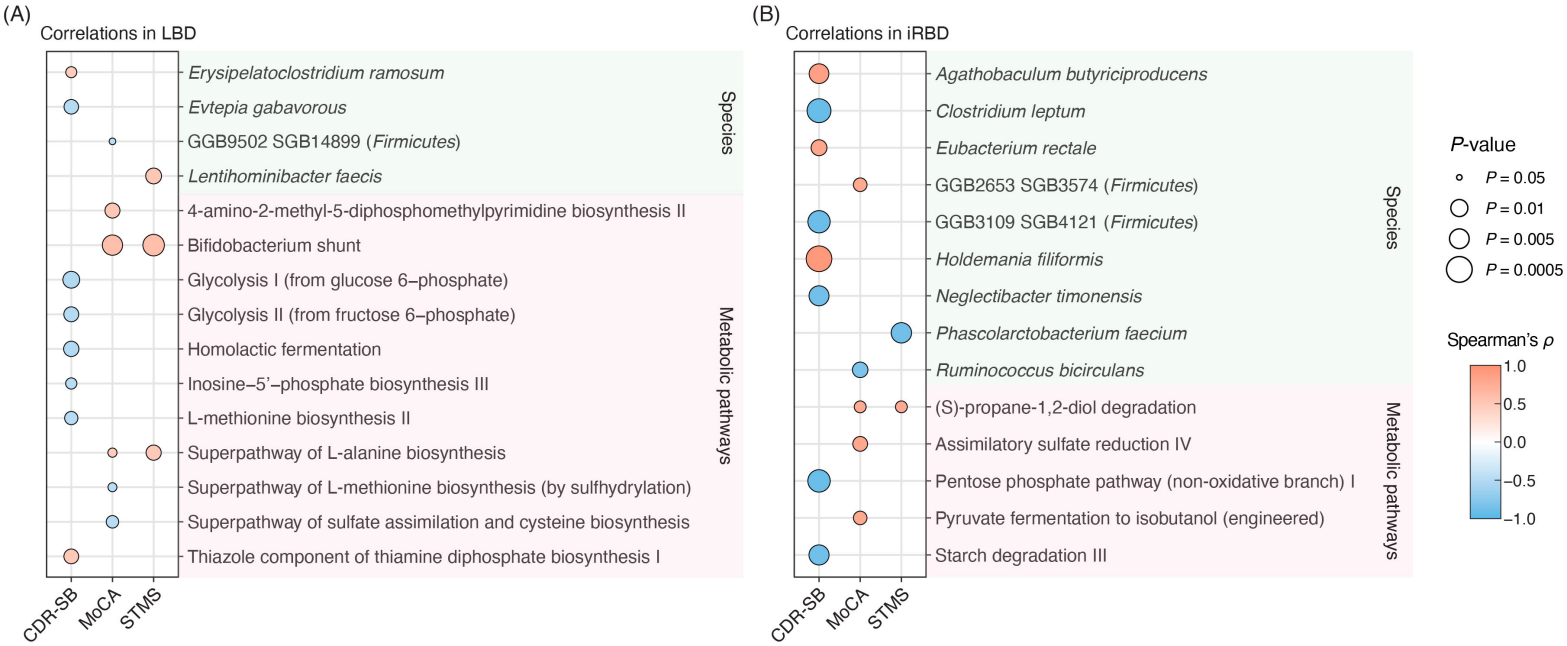
Correlations between clinical measures and gut microbial species and pathways in **(A)** LBD and **(B)** iRBD. Spearman partial correlations adjusting for age and BMI and corresponding *P*-values were calculated for each clinical measure and microbial features. Each bubble represents a correlation meeting both significance (*P* < 0.05) and effect size (|*ρ*| > 0.4) thresholds. Positive correlations are shown in red and negative in blue. Bubble size reflects *P*-value magnitude, with larger bubbles indicating smaller *P*-values. Correlations were further validated by permutation testing (1,000 iterations, random subsampling without replacement); empirical *P* < 0.05 required for inclusion. CDR-SB, Clinical Dementia Rating Sum of Boxes; MoCA, Montreal Cognitive Assessment; STMS, Short Test of Mental Status. All *P*-values are unadjusted for multiple comparisons.

We calculated Spearman partial correlation coefficients (*ρ*) and corresponding *P*-values between four clinical features (CDR-SB, MoCA, STMS, and MDS-UPDRS III) and the abundances of microbial species and metabolic pathways (**Supplementary Tables 13**–**16**). Only features meeting both the primary significance criteria (unadjusted *P* < 0.05 and |*ρ*| > 0.4) and resampling-based validation (unadjusted empirical *P* < 0.05) are reported (**Figures 7A, B**; **Supplementary Tables 17** and **18**). Among these clinical scores, CDR-SB, MoCA, and STMS reflect dementia severity and cognitive performance: higher CDR-SB values indicate more severe dementia, whereas lower MoCA and STMS scores indicate greater cognitive impairment. MDS-UPDRS III measures motor symptom severity, with higher scores indicating worse motor function.

In LBD patients, several microbial metabolic pathways were positively associated with better cognitive performance and less severe dementia. The Superpathway of L-alanine biosynthesis and the Bifidobacterium shunt were positively correlated with both MoCA and STMS scores. Homolactic fermentation, Glycolysis I (from fructose 6-phosphate), and Glycolysis II (from fructose 6-phosphate) were negatively correlated with CDR-SB, and 4-amino-2-methyl-5-diphosphomethylpyrimidine biosynthesis II was positively correlated with MoCA (**Figure 7A**). All pathways described above also showed negative correlations with MDS-UPDRS III scores (**Supplementary Table 15**). At the species level, *Erysipelatoclostridium ramosum* was positively correlated with CDR-SB, while *Evtepia gabavorous* was negatively correlated with CDR-SB. *Lentihominibacter faecis* was positively correlated with STMS (**Figure 7A**). Unlike the pathway-level findings, these species associations were each specific to a single clinical measure.

In iRBD patients, several microbial features also correlated with better cognitive outcomes and less severe dementia. *Clostridium leptum*, *Neglectibacter timonensis*, and Starch degradation III were negatively correlated with CDR-SB (**Figure 7B**). Additionally, (S)-propane-1,2-diol degradation was positively correlated with both MoCA and STMS (**Figure 7B**).

## Discussion

In this study, we identified alterations in gut microbiome taxonomy and functional potential across the LBD continuum, spanning the prodromal stage of iRBD through clinical manifestation of LBD. By integrating shotgun metagenomic sequencing with a household-matched study design, our analysis moves beyond community-level descriptions to reveal convergent taxonomic and metabolic potential shifts that are not apparent from global diversity metrics alone. Collectively, our findings suggest that subtle but consistent changes in microbial functions are shared across both the prodromal and symptomatic stages of the LBD continuum and may be relevant to disease progression through gut-brain axis mechanisms. These findings are presented as exploratory and hypothesis-generating, and are intended to provide specific, testable targets for future validation studies in larger cohorts.

At the level of overall community structure, neither α-diversity nor β-diversity differed significantly between patients and cohabitant controls. These findings are consistent with prior studies that utilized household-matched designs and support that broad measures of diversity may be insensitive to disease-associated changes when environmental exposure and dietary confounders are carefully controlled (Song et al., 2013; Abeles et al., 2016; Teigen et al., 2024). Importantly, however, the absence of global diversity shifts does not imply functional equivalence. Rather, it highlights a potential strength of our study: the ability to detect fine-grained, functionally relevant microbial alterations that may occur within otherwise stable community structures.

To highlight the most informative microbial alterations, the discussion focuses on microbial species and pathways that showed both statistical significance (unadjusted *P* < 0.05) and meaningful effect size (|Cohen’s *d*| > 0.4). Microbial features lacking species-level characterization are not interpreted in depth. In some cases, features with more modest rankings are included because they show convergent signals across the LBD continuum or have established relevance to the disease.

One finding from our differential abundance analyses is the increased abundance of microbial taxa potentially associated with impaired gut barrier integrity. We observed higher abundance of *Hungatella hathewayi* (unadjusted *P* = 0.015, Cohen’s *d* = 0.477) in LBD and *Collinsella aerofaciens* (unadjusted *P* < 0.001, Cohen’s *d* = 1.202) in iRBD compared with their cohabitant controls. *H. hathewayi* has been shown to degrade glycosaminoglycans (GAGs) (Rawat et al., 2022), which are key structural components supporting gut epithelial barrier integrity (Francis et al., 2024). Experimental studies have suggested that excessive depletion of GAG-rich layers increases tissue permeability and weakens barrier function (Hurst et al., 2016). In this context, increased abundance of this species may therefore be associated with altered gut integrity and low-grade inflammation. Similarly, *C. aerofaciens* and the *Collinsella* genus have previously been reported to be more abundant in RBD (Huang et al., 2023), early PD (Huang et al., 2023), and DLB (Nishiwaki et al., 2022). Beyond neurodegenerative disease, *Collinsella* has also been found to be more abundant in rheumatoid arthritis, where it has been shown to stimulate IL-17A production, which is a cytokine linked to increased intestinal permeability and promotes chronic inflammation (Chen et al., 2016). Therefore, our findings suggest that the increased abundance of these taxa in LBD and iRBD may be associated with gut barrier dysfunction and systemic inflammatory signaling, though this requires functional confirmation in future studies.

In addition, we observed higher abundance of microbial pathways associated with putative LPS biosynthesis in both disease groups. Specifically, the ADP-L-glycero-β-D-manno-heptose biosynthesis pathway (unadjusted *P* = 0.046, Cohen’s *d* = 0.461) was enriched in LBD, while the O-antigen building blocks biosynthesis pathway (unadjusted *P* = 0.002, Cohen’s *d* = 1.407) was elevated in iRBD. Both pathways contribute essential components to LPS assembly: ADP-L-glycero-β-D-manno-heptose serves as a precursor for the inner core (Kneidinger et al., 2002; Whitfield et al., 2020), and O-antigen building blocks form the outer polysaccharide domain of LPS (Bertani and Ruiz, 2018; Whitfield et al., 2020). Elevated microbial potential for LPS biosynthesis may be relevant to α-synucleinopathies through several proposed mechanisms. First, LPS has been reported to be associated with compromised gut epithelial integrity and increased intestinal permeability (Guo et al., 2013; Guo et al., 2015; Nighot et al., 2017), thereby potentially facilitating exposure of the enteric nervous system to microbial antigens (van Kessel and El Aidy, 2019). Moreover, *in vitro* studies suggest that LPS can promote α-synuclein aggregation in the gut (Bhattacharyya et al., 2019; Bhattacharyya and Bhunia, 2021). Overall, the increased abundance of LPS biosynthesis related pathways observed in both LBD and iRBD may be relevant to mechanisms that have been associated with heightened intestinal permeability and potential α-synuclein aggregation. One hypothesis is that this aggregation in the gut may facilitate “prion-like” propagation of α-synuclein through the enteric nervous system and ascend to the brainstem through the vagus nerve, as proposed in Braak’s staging schema (Visanji et al., 2013). However, whether this reflects active LPS production or downstream consequences for neurodegeneration remains to be directly tested.

We next examined microbial taxa and functional pathways associated with putative SCFA production, which are particularly sensitive to dietary and environmental influences. SCFAs have been reported to play important roles in maintaining gut barrier integrity (Peng et al., 2009), exerting anti-inflammatory effects (Segain et al., 2000), and regulating neurotransmission (Rios-Covian et al., 2017; Deleu et al., 2021; Facchin et al., 2024). We identified a significant reduction in the starch degradation III pathway (unadjusted *P* = 0.012, Cohen’s *d* = –0.417) in LBD, accompanied by decreased abundance of multiple gene families across several enzymatic steps, suggesting reduced microbial capacity for starch degradation. The terminal products of this pathway feed into glycolysis and are metabolized to pyruvate, which can be further converted to acetate (Flint et al., 2012; Louis and Flint, 2017). Complementing this finding, the β-(1,4)-mannan degradation pathway (unadjusted *P* = 0.013, Cohen’s *d* = –0.593) also had lower abundance in LBD group compared with their cohabitant controls. β-(1,4)-mannan is a hemicellulose that functions as a dietary fiber (Gropper et al., 2009). The end products of this pathway also feed into glycolysis and can be converted to acetate (Caspi et al., 2018). Acetate has been reported to cross the blood-brain barrier and may serve as a preferred energy source for astrocytes with proposed roles in supporting glutamate and GABA homeostasis (Waniewski and Martin, 1998; Frost et al., 2014). Within the brain, acetate is converted to acetyl-CoA, a key substrate for histone acetylation that has been linked to memory-related gene expression (Soliman and Rosenberger, 2011; Mews et al., 2017). Thus, reduced complex carbohydrate degradation potential in LBD may be associated with reduced acetate availability with possible downstream implications for compromised metabolic and epigenetic processes along the gut–brain axis, although this remains to be directly tested. In addition, several known SCFA-producing taxa, including *Roseburia hominis* (unadjusted *P* = 0.001, Cohen’s *d* = –0.692), *Eubacterium ramulus* (unadjusted *P* = 0.006, Cohen’s *d* = –0.569), and *Intestinimonas butyriciproducens* (unadjusted *P* = 0.013, Cohen’s *d* = –0.533), were decreased in LBD, while *Bacteroides* were reduced in both LBD (unadjusted *P* = 0.033, Cohen’s *d* = –0.491) and iRBD (unadjusted *P* = 0.001, Cohen’s *d* = –1.305). The consistent loss of these beneficial commensals across disease stages suggests a shared microbial pattern that may be associated with weakened gut barrier integrity and disrupted gut-brain signaling from the prodromal through symptomatic phases.

Finally, we identified differences in microbial pathways related to putative neurotransmitter metabolism, particularly the L-histidine degradation III pathway, which was reduced in both LBD (unadjusted *P* = 0.027, Cohen’s *d* = –0.498) and iRBD (unadjusted *P* < 0.001, Cohen’s *d* = –1.921) relative to controls. Multiple gene families involved in this pathway showed lower abundance in disease groups. In this pathway, L-histidine is converted to L-glutamate, which can enter the GABA shunt pathway and be converted to GABA by glutamate decarboxylase. Among the significant gene families mapped to glutamate decarboxylase, both in LBD and one of three in iRBD showed higher abundance in controls; this suggests a possible progressive decline in microbial potential to convert L-glutamate to GABA from iRBD to LBD. Increasing evidence suggests that gut-derived GABA can influence circulating and central nervous system GABA levels, suggesting gut microbiome as a potential contributor to GABA-related neural signaling (Strandwitz, 2018; Braga et al., 2024). Consistent with this, previous studies have reported increased abundance of GABA-consuming taxa and enrichment of GABA degradation pathways in Parkinson’s Disease (Wallen et al., 2022; Marzouk et al., 2025). Additionally, a recent study has found that probiotic and prebiotic intervention restored brain GABA levels and reduced motor impairment and neuronal loss in animal models (Zhong et al., 2023). Furthermore, impaired GABA neurotransmission has been implicated in REM sleep without atonia, the neurophysiologic substrate of RBD (Brooks and Peever, 2011). Taken together, the reduced abundance of microbial pathways and gene families related to L-glutamate production and its conversion to GABA in both iRBD and LBD may link to alterations in microbial functional potential associated with GABA-related metabolic processes, which could have implications for disrupted excitatory-inhibitory balance along the gut-brain axis. These changes could be associated with RBD symptom development or neurodegenerative processes across the LBD spectrum, although further mechanistic studies are needed to clarify these relationships.

Several limitations should be acknowledged when interpreting the results. First, we were unable to adjust for sex in the statistical models due to strong collinearity between sex and case-control status, which is a common challenge in studies of LBD and iRBD given their marked male predominance. As a result, including sex as a covariate in mixed-effects models would not meaningfully disentangle sex effects from disease status and could obscure true disease-associated microbial signals. Second, although cohabitant controls were included to minimize environmental confounding, we did not directly quantify the degree of shared dietary intake within households. Consequently, residual dietary influences may not have been fully accounted for. Nevertheless, the use of cohabitant controls remains a major strength of this study, as it substantially reduces unmeasured environmental and lifestyle variability compared with designs relying on unrelated controls. Third, the relatively small sample size, particularly for the iRBD group and their matched controls (n = 10), limits the statistical power to detect small or moderate effects and is unlikely to be fully representative of the broader population. Future studies with larger, multi-center cohorts will be necessary to validate and extend these observations. Despite these constraints, the consistent directional patterns observed across both LBD and iRBD relative to their household-matched controls lend confidence that the identified alterations reflect biologically relevant signals rather than chance findings. Fourth, we did not apply multiple testing correction in the primary analysis, as very few results remained statistically significant after Benjamini–Hochberg false discovery rate adjustment given the large number of simultaneous comparisons across species, pathways, and gene families. Consequently, the reported results are subject to an increased risk of false-positive findings and should be interpreted as hypothesis-generating and exploratory rather than biologically conclusive (Rothman, 1990). Nevertheless, our results can provide a focused set of microbial species and pathways that may serve as candidates for validation in larger, independent cohorts (Rothman, 1990). Fifth, a key limitation of this study is the compositional nature of relative abundance data in microbiome analyses (Gloor et al., 2017). Total sum scaling forces all microbial features to sum to a constant value (i.e., 100%), meaning that changes in one taxon, pathway, or gene family influences the relative abundance of others. As a result, some observed differences may reflect compositional artifacts rather than true biological changes. In this study, we applied an arcsine square-root transformation to improve data distribution and stabilize variance for statistical analyses. While this transformation helps meet statistical assumptions, it does not resolve the underlying compositional issue, and our results should therefore be interpreted with appropriate caution. Finally, this study is cross-sectional, which limits the ability to infer causal relationships between gut microbial alterations and disease status. The observed associations with functions such as gut barrier integrity, neuroinflammation, and α-synuclein aggregation should therefore be interpreted with caution. Nevertheless, the consistent alterations identified across two stages of the LBD continuum provide a framework for future longitudinal and mechanistic studies. To this point, the candidate signals reported here define a clear and specific agenda for validation in larger, independent cohorts using complementary methodologies such as targeted qPCR, metatranscriptomics, and metabolomics.

## Conclusions

Our findings suggest that the LBD disease continuum may be associated with changes in specific microbial taxa and metabolic pathways, rather than global disruption of the overall gut microbiome. These changes include reduced abundance of pathways related to complex carbohydrate fermentation in LBD and neurotransmitter-related metabolism in both LBD and iRBD, as well as increased abundance of taxa potentially associated with gut barrier disruption and pathways related to LPS biosynthesis in both disease groups. Overall, our findings support the possibility that gut microbial changes may accompany neurodegenerative processes along the disease continuum. By focusing on biologically interpretable taxonomic and metabolic features rather than broad diversity metrics alone, this study provides a framework for future longitudinal and mechanistic studies investigating potential links between the gut microbiome and LBD pathogenesis and progression.

## Data Availability

Sequencing data for stool metagenomes used in this study have been deposited at NCBI’s SRA data repository (PRJNA1393457) and can be downloaded without any restrictions at https://www.ncbi.nlm.nih.gov/bioproject/PRJNA1393457/. The deposited sequences include .fastq files for 70 stool metagenomes collected from 25 LBD patients, 10 iRBD patients, as well as their 35 cohabitant controls. Human reads were identified and removed prior to data upload. Code used for data analysis is available in the following GitHub repository: https://github.com/xiaowei-zhao-1111/LBD_Gut_Microbiome_2026.
