# Supplementary material for: Shotgun metagenomic analysis reveals taxonomic and functional alterations in the gut microbiome across prodromal and symptomatic Lewy body disease
Source: Front Microbiomes. 2026 Jul 15;5:1834726. doi: 10.3389/frmbi.2026.1834726 (PMC13416100; doi:10.3389/frmbi.2026.1834726)
Supplement: Supplementary file 1 [file DataSheet1.pdf]

Supplementary Figures 1–5

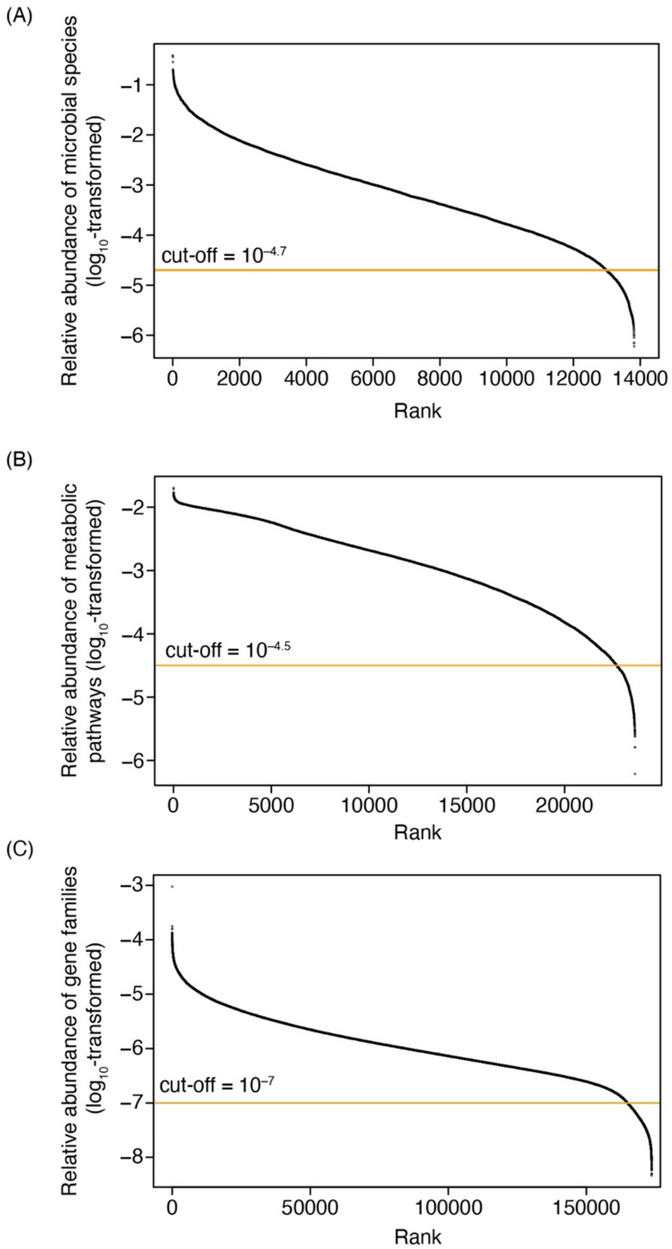

Supplementary Figure 1. Rank-abundance plots used to define presence filtering thresholds for microbial species, metabolic pathways, and gene families. Rank abundance curves for species **(A)**, metabolic pathways **(B)**, and gene families **(C)**, with each black point representing the relative abundance ordered from highest to lowest. The horizontal orange lines indicate the thresholds used to remove extremely low abundance features to minimize the influence of noise and technical artifacts. That is, values below these cut-offs were set to zero.

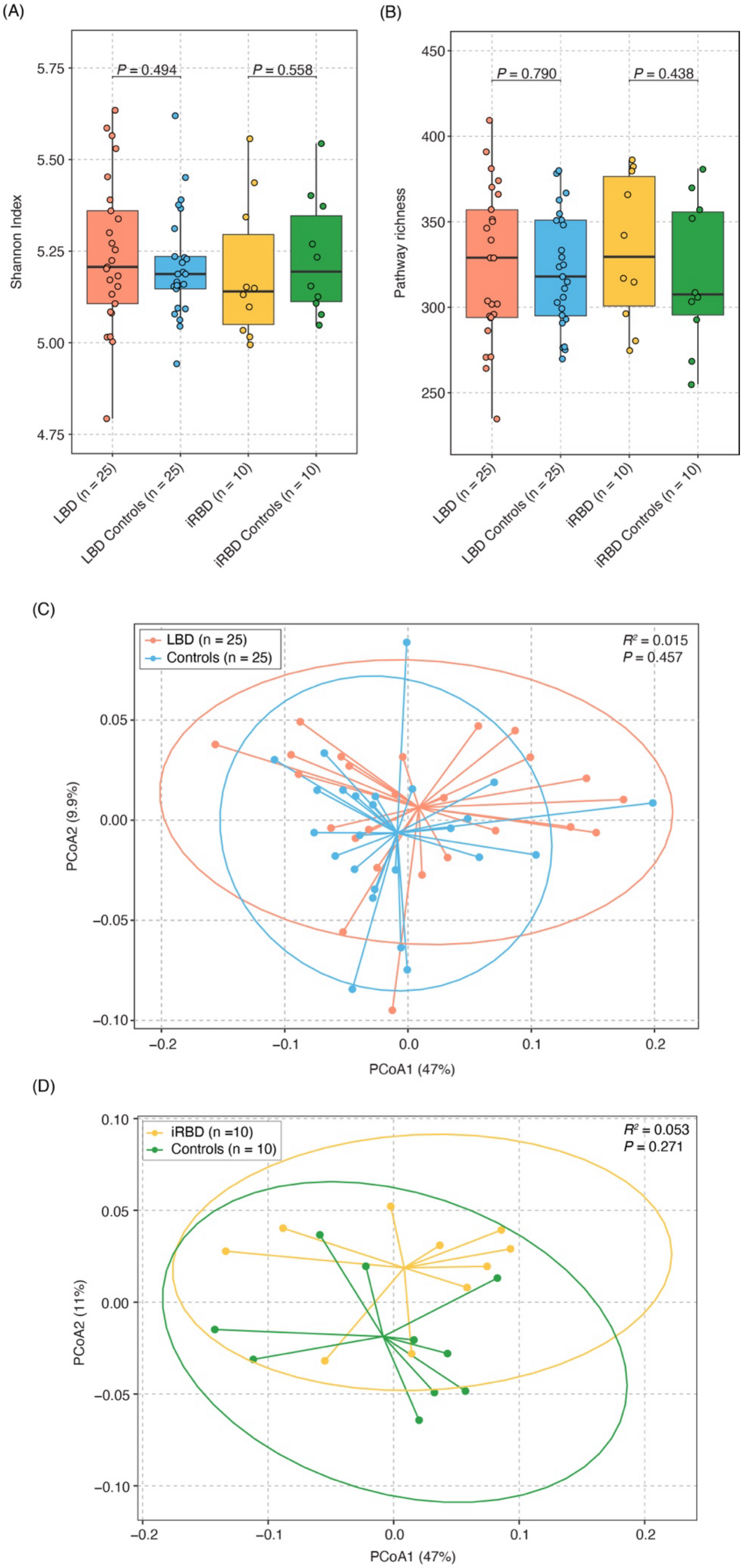

(A)
P = 0.494
P = 0.558
Shannon Index
5.75
5.50
5.25
5.00
4.75
LBD (n = 25)
LBD Controls (n = 25)
iRBD (n = 10)
iRBD Controls (n = 10)
(B)
P = 0.790
P = 0.438
Pathway richness
450
400
350
300
250
LBD (n = 25)
LBD Controls (n = 25)
iRBD (n = 10)
iRBD Controls (n = 10)
(C)
LBD (n = 25)
Controls (n = 25)
R² = 0.015
P = 0.457
PCoA2 (9.9%)
PCoA1 (47%)
0.05
0.00
−0.05
−0.10
−0.2
−0.1
0.0
0.1
0.2
(D)
iRBD (n =10)
Controls (n = 10)
R² = 0.053
P = 0.271
PCoA2 (11%)
PCoA1 (47%)
0.10
0.05
0.00
−0.05
−0.10
−0.2
−0.1
0.0
0.1
0.2

Supplementary Figure 2. α- and β-diversity analysis based on metabolic pathway relative abundance. (A–B) No significant differences in metabolic pathway-level Shannon index or richness were observed between disease and their cohabitant controls. Statistical comparisons between LBD or iRBD and controls were performed using mixed-effects linear regression models with household ID as a random effect (to account for intra-household correlation). **(C–D)** Principal coordinate analysis (PCoA) plots based on species-level Bray–Curtis dissimilarity showed no clear separation by case-control status, and permutational multivariate analysis of variance (PERMANOVA) tests indicated that disease diagnosis explained minimal variation in overall microbial composition.

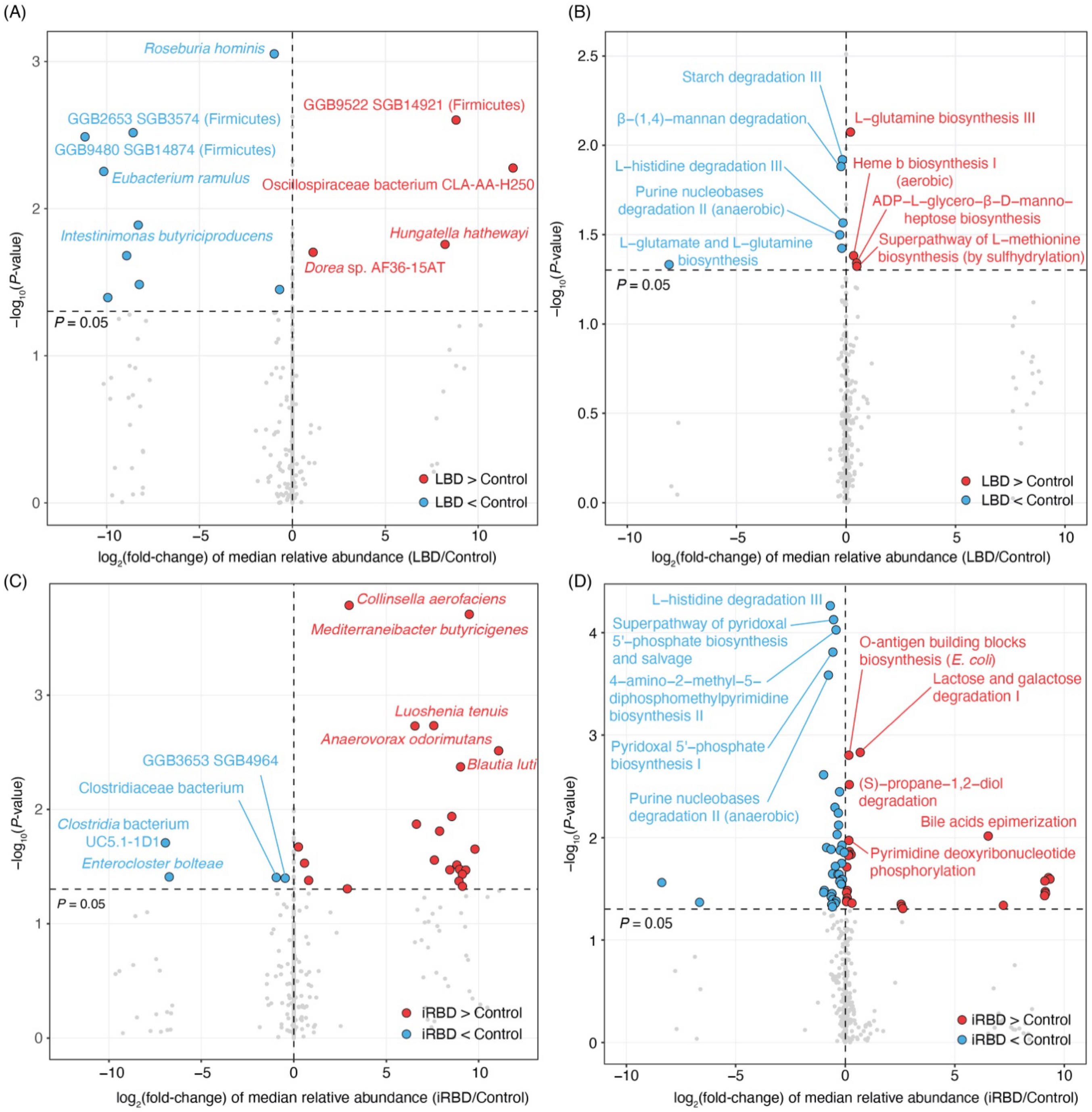

Supplementary Figure 3. Differentially abundant microbial species and metabolic pathways in LBD and iRBD compared with cohabitant controls. Volcano plots display $\log_2$(fold-change) in median relative abundance (x-axis) vs. statistical significance ($-\log_{10}P$-value, y-axis) for microbial species and metabolic pathways in comparisons of LBD vs. controls **(A, B)** and iRBD vs. controls **(C, D)**. Statistical significance was defined as $P < 0.05$. Features with positive $\log_2$(fold-change) are more abundant in the disease group, while those with negative values are more abundant in controls. Up to five features with the largest effect sizes on either side of the plot are labeled for clarity.

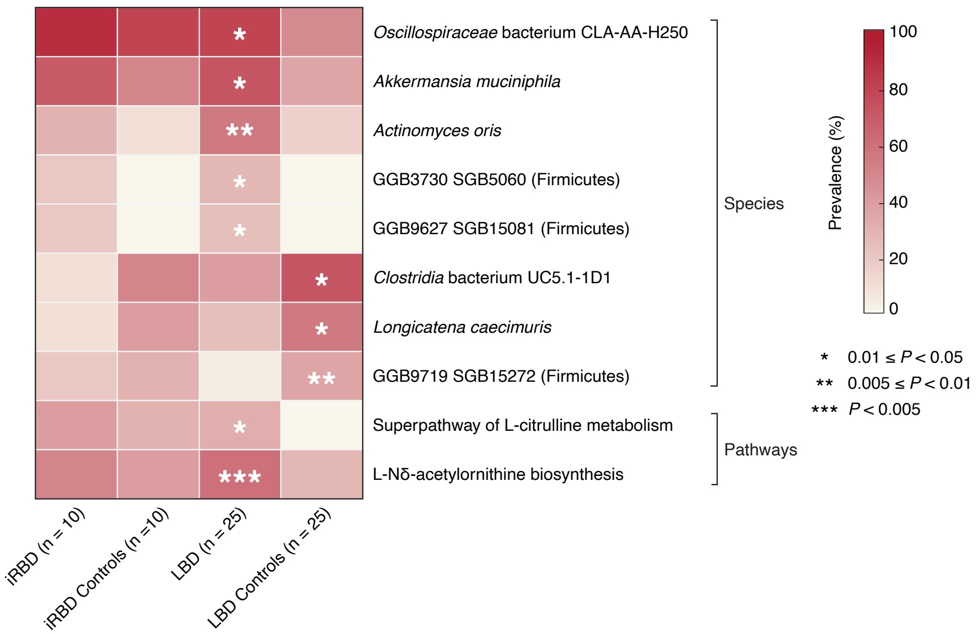

Supplementary Figure 4. Progressive alterations in microbial species and pathways along the Lewy body disease continuum. The heatmap shows the prevalence of microbial species and metabolic pathways that differed significantly between LBD patients and their cohabitant controls. For each feature, the prevalence pattern in iRBD and its matched controls is also shown, revealing similar directional trends that did not reach statistical significance. Color intensity reflects the proportion of individuals in each group carrying the corresponding feature. *P*-values were obtained using the Fisher's exact test. *, $0.01 \le P < 0.05$; **, $0.005 \le P < 0.01$; ***, $P < 0.005$.

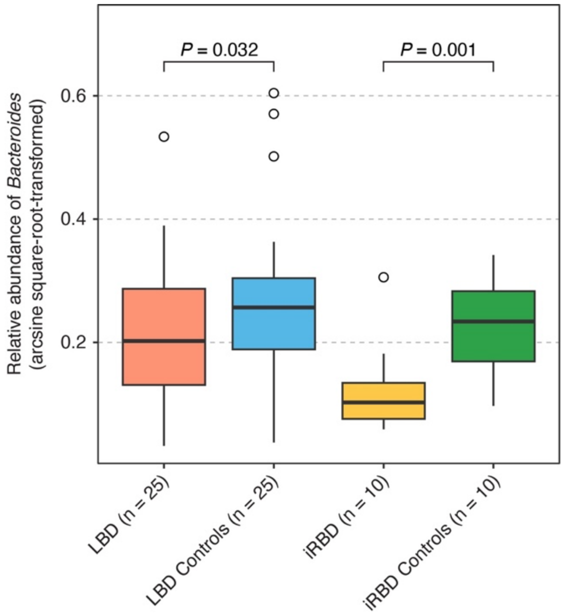

Supplementary Figure 5. Relative abundance of *Bacteroides* in LBD and iRBD compared with cohabitant controls. Boxplots show the arcsine square-root-transformed relative abundance of *Bacteroides* in LBD patients, iRBD patients, and their respective household cohabitants. *P*-values were obtained using mixed-effects linear regression models adjusted for age and sex, with household ID included as a random effect. Both LBD and iRBD groups displayed significantly lower *Bacteroides* abundance compared with their cohabitant controls.
